# Hepatotoxicity in patients with solid tumors treated with PD-1/PD-L1 inhibitors alone, PD-1/PD-L1 inhibitors plus chemotherapy, or chemotherapy alone: systematic review and meta-analysis

**DOI:** 10.1007/s00228-020-02903-2

**Published:** 2020-06-08

**Authors:** Xiaodi Guo, Wendong Li, Jiexuan Hu, Emily C. Zhu, Qiang Su

**Affiliations:** 1 Department of Oncology, Beijing Ditan Hospital, Capital Medical University, Beijing, 100015 China; 2 Department of Oncology, Beijing Friendship Hospital, Capital Medical University, Beijing, 100050 China; 3 Department of CIS & Quantitative Methods, McCoy College of Business, Texas State University, San Marcos, TX USA

**Keywords:** Hepatotoxicity, PD-1/PD-L1 inhibitors, Chemotherapy, Meta-analysis, Solid tumors

## Abstract

**Background:**

This meta-analysis examined the risk of hepatotoxicity in patients with solid tumors who received a PD-1/PD-L1 inhibitor alone, a PD-1/PD-L1 inhibitor plus chemotherapy, or chemotherapy alone.

**Methods:**

Potentially eligible studies were identified by searches of Embase and PubMed. All included studies were randomized controlled trials (RCTs) that examined patients with solid tumors who received a PD-1/PD-L1 inhibitor and/or chemotherapy.

**Results:**

We included 20 clinical trials (11,634 patients). Thirteen trials compared PD-1/PD-L1 inhibitor monotherapy with chemotherapy. These two groups had similar risk for elevated markers of hepatotoxicity (based on analysis of all marker grades and high marker grades), although the PD-1/PD-L1 inhibitor group had an elevated relative risk (RR) of elevated aspartate aminotransferase (AST; RR = 2.13, 95% CI = 1.04 to 4.36, *P* = 0.04) when considering high grades alone; however, this disparity was not significant for comparisons of the pembrolizumab and nivolumab subgroups with the chemotherapy group. Compared with chemotherapy, PD-1/PD-L1 inhibitors increased the risk of all-grade hepatitis (RR = 5.85, 95% CI = 1.85 to 18.46, *P* < 0.01), and high-grade hepatitis (RR = 5.66, 95% CI = 1.58 to 20.27, *P* < 0.01). Seven other studies compared PD-1/PD-L1 inhibitor plus chemotherapy with chemotherapy alone. The combined treatment led to a higher risk for all-grade hepatitis (RR = 2.14, 95% CI = 1.29 to 3.55, *P* < 0.01) and high-grade hepatitis (RR = 5.24, 95%CI = 1.89 to 14.52, *P* < 0.01), but these groups had similar risk for all-grade and high-grade elevated markers of hepatotoxicity.

**Conclusions:**

Relative to chemotherapy alone, PD-1/PD-L1 inhibitors with or without chemotherapy increased the risk of all-grade and high-grade hepatitis, but generally did not increase the risk of elevated blood markers of hepatotoxicity.

**Electronic supplementary material:**

The online version of this article (10.1007/s00228-020-02903-2) contains supplementary material, which is available to authorized users.

## Introduction

Cancer has been a leading cause of mortality among people younger than 70 years old for more than 50 years [1]. Although cancer treatments are expensive throughout the world, there are limited effective therapeutic approaches for advanced malignancies. Immune checkpoint inhibitors (ICIs) are the most recent breakthrough in the treatment of cancer, and these agents have dramatically increased the therapeutic options for treatment of multiple cancers. ICIs are now the fourth pillar of cancer treatment, in addition to surgery, radiotherapy, and chemotherapy.

The era of ICIs started in 2011 when the US Food and Drug Administration (FDA) approved the new anticancer drug ipilimumab, a fully human IgG1 antibody that blocks the cytotoxic T lymphocyte-antigen-4 (CTLA-4) on the surface of T lymphocytes. Subsequently, the FDA approved pembrolizumab and nivolumab, the first two programmed cell-death 1 (PD-1) inhibitors, and then atezolizumab, durvalumab, and avelumab, inhibitors of programmed death-ligand 1 (PD-L1). Clinical oncologists now use all of these agents to treat multiple malignancies [2]. Oncologists currently believe that during the development of cancer, an individual’s immune system becomes “tolerant” of cancer cells and that alterations of immune checkpoint pathways downregulate immune functions so that cancer cells can proliferate. The ICIs inhibit the inactivation of T lymphocytes and thereby enhance anticancer and cytotoxic effects.

However, by activating the immune system, ICIs can also cause excessive immune reactions against healthy normal organs, known as immune-related adverse events (irAEs) [3]. Specifically, ICIs can cause a variety of hepatic toxicities, ranging from a mild increases of transaminase levels to life-threatening liver failure. Other cancer treatments can also lead to hepatic toxicities. For example, chemotherapeutic agents are commonly implicated in the etiology of drug-induced liver injury (DILI) [4]. Two recent meta-analyses [5, 6] examined the risk of hepatotoxicity associated with ICIs using control groups that received placebo, biologic agents, other ICIs, or chemotherapy. In contrast, the present meta-analysis focuses on liver damage and toxicity in patients who received PD-1/PD-L1 inhibitors alone or in combination with chemotherapy. The combined treatment of patients with anti-PD-1/PD-L1 agents and chemotherapy has become increasingly prevalent. However, the effect of this more aggressive treatment on the risk and severity of hepatotoxicity relative to chemotherapy alone remains unknown.

The present systematic review and meta-analysis investigated the relative risk of hepatotoxicity in patients with solid tumors who received an anti-PD-1/PD-L1 agent alone, an anti-PD-1/PD-L1 agent with chemotherapy, or standard chemotherapy alone.

## Methods

This systematic review and meta-analysis was conducted according to the guidelines of the Cochrane Handbook for Systematic Review of Interventions [7], and the results were reported according to the PRISMA Statement [8].

### Search strategy

PubMed and Embase databases were searched for randomized clinical trials (RCTs) using the following key words: “PD-1”, “PD-L1”, “nivolumab”, “pembrolizumab”, “atezolizumab”, “durvalumab”, and “avelumab” for publications on or before September 11, 2019 (Supplementary Table S1).

### Inclusion and exclusion criteria

All eligible studies were RCTs of humans with solid tumors; had at least one control arm consisting of standard chemotherapy; compared a PD-1/PD-L1 inhibitor vs*.* chemotherapy or a PD-1/PD-L1 inhibitor plus chemotherapy vs*.* the same chemotherapy agent (with or without placebo); and measured at least one marker of hepatotoxicity in both study arms (alanine aminotransferase [ALT], aspartate aminotransferase [AST], bilirubin [BIL], alkaline phosphatase [ALP], gamma-glutamyl transferase [GGT], and hepatitis).

Studies were excluded if they were phase I trials, single-arm studies, or trials where patients had no adverse hepatic events in either treatment arm; if patients received other agents simultaneously, such as other ICIs and targeted drugs; if they were retrospective studies, meeting abstracts, case reports, unfinished studies, duplicate reports, letters, or reviews; and if they were in any language other than English.

### Data extraction

Two authors (XG and QS) independently evaluated all studies for eligibility by initially checking the titles, abstracts, and full texts of the studies following the patient, intervention, comparison, and outcome (PICO) chart [9]. The following information was extracted from all eligible studies: first author’s last name, year of publication, trial phase, treatment groups, primary endpoint, underlying solid malignancy, number of patients in each group, chemotherapy agents, and adverse events (AEs). The two categories of AEs were all grade (1 to 5) and high-grade (3 to 5) liver AEs [10], namely increased ALT, AST, BIL, ALP, GGT, and hepatitis. In these RCTs, hepatitis was limited to immune-mediated or immune-related AEs.

### Data analysis

The risk of bias was assessed using Review Manager 5.3 software (Cochrane Collaboration 2014, Nordic Cochrane Center, Copenhagen, Denmark). Two authors (XG and QS) independently assessed the quality of the included RCTs using the Cochrane risk of bias tool [11]. Relative risk (RR) and 95% CIs of all-grade and high-grade hepatotoxicity events were the principal measures. Data analysis was performed using R software (version 3.6.1) with the meta-package.

Heterogeneity among the RCTs was quantified using the *Q* test and *I*^2^ statistics. If the *I*^2^ value was less than 50%, a fixed-effects model was used; otherwise, a random-effects model was used [12, 13]. Sensitivity analysis was performed by removing one study at a time and recalculation of the results. Potential publication bias was evaluated by Begg’s test and Egger’s test. All *P* values were 2-tailed, and *P* value below 0.05 was considered significant.

## Results

### Literature search

Our initial search of PubMed and Embase yielded 1884 potentially relevant clinical trials. After removal of matching studies from the two databases and review of the titles and abstracts, we initially excluded 1733 studies because they did not fulfill our criteria. The excluded studies included review articles, retrospective studies, case reports, phase I/II trials, single-arm studies, non-randomized clinical trials, and studies of non-solid tumors. Further review led to the exclusion of 124 studies because they used combinations of different ICIs or compared an ICI with an intervention other than chemotherapy. After review of the full-text of the remaining 27 studies, we excluded 7 trials because they had no information related to hepatotoxicity (Supplementary Figure S1). The 20 eligible studies examined patients with non-small cell lung cancer (NSCLC, *n* = 11), melanoma (n = 3), carcinoma of the head and neck (*n* = 2), and small cell lung cancer, gastro-esophageal junction cancer, urothelial carcinoma, and breast cancer (1 each). None of the included chemotherapy-controlled RCTs examined durvalumab. The 20 studies in this meta-analysis examined 11,634 patients.

Thirteen of the 20 studies examined PD-1/PD-L1 inhibitor monotherapy vs. chemotherapy alone, and the other 7 studies examined PD-1/PD-L1 inhibitor plus chemotherapy vs. chemotherapy alone. We did not include any of the PD-1/PD-L1 monotherapy studies that examined atezolizumab, because none of them reported hepatic AEs in the chemotherapy arm. The 13 studies of PD-1/PD-L1 inhibitor monotherapy vs. chemotherapy examined patients treated with nivolumab (6 studies, 1395 patients), pembrolizumab (6 studies, 2481 patients), and avelumab (1 study, 393 patients). The 7 studies of PD-1/PD-L1 inhibitor plus chemotherapy vs. chemotherapy examined patients treated with atezolizumab (4 studies, 1516 patients) and pembrolizumab (3 studies, 742 patients).

Tables 1 and 2 show the baseline details and the relevant all-grade and high-grade hepatotoxic AEs in each trial. Analysis using the Cochrane risk of bias tool indicated a low risk of bias for all included studies (Supplementary Figure 2). In this analysis, we graded all laboratory values according to the National Cancer Institute Common Terminology Criteria for Adverse Events (CTCAE) version 4.0 (Supplementary Table S2).

**Table 1:** Characteristics of the 13 randomized controlled trials that compared PD-1/PD-L1 inhibitors *vs.* chemotherapy

References	Trial Phase	Tumor type	Endpoint	Treatment arms	Pts	Increased ALT		Increased AST		Hepatitis		Increased BIL		Increased ALP		Increased GGT	
						G1-5	G3-5	G1-5	G3-5	G1-5	G3-5	G1-5	G3-5	G1-5	G3-5	G1-5	G3-5
[14]	III	Melanoma	OS,ORR	Niv 3 mg/kg q2w	268	7	2	11	1	NA	NA	NA	NA	NA	1	NA	NA
				ICC q3w	102	1	0	2	0	NA	NA	NA	NA	NA	NA	NA	NA
[15]	III	Non-squamous NSCLC	OS	Niv 3 mg/kg q2w	287	9	0	9	1	NA	NA	1	0	2	0	2	2
				Doc 75 mg/m^2^ q3w	268	4	1	2	0	NA	NA	0	0	4	1	0	0
[16]	III	squamous-cell NSCLC	OS	Niv 3 mg/kg q2w	131	2	0	2	0	NA	NA	0	0	NA	NA	NA	NA
				Doc 75 mg/m^2^ q3w	129	1	1	1	1	NA	NA	1	0	NA	NA	NA	NA
[17]	III	First-line NSCLC	PFS	Niv 3 mg/kg q2w	267	19	7	23	7	NA	NA	NA	NA	NA	NA	NA	NA
				Platinum-based ICC q3w	263	14	2	12	1	NA	NA	NA	NA	NA	NA	NA	NA
[18]	III	Head + neck	OS	Niv 3 mg/kg q2w	236	2	1	2	0	NA	NA	1	0	2	0	0	0
				Methotrexate/Doc	111	3	1	2	0	NA	NA	0	0	0	0	1	1
[19]	III	Melanoma	OS	Niv 3 mg/kg q2w	206	3	2	2	1	NA	NA	2	0	NA	NA	NA	NA
				Dac 1 g/m^2^ q3w	205	3	1	4	1	NA	NA	1	0	NA	NA	NA	NA
[20]	III	Head + neck	OS	Pem 200 mg q3w	246	5	0	3	1	2	1	2	1	1	0	1	0
				Methotrexate/Doc	234	8	3	8	3	0	0	4	0	4	0	2	1
[21]	III	First-line NSCLC	OS	Pem 200 mg q3w	636	45	9	41	4	9	7	NA	NA	NA	NA	NA	NA
				Platinum-based ICC q3w	615	53	5	42	2	0	0	NA	NA	NA	NA	NA	NA
[22]	III	GC/GEJC	OS,PFS	Pem 200 mg q3w	294	NA	NA	NA	NA	4	4	NA	NA	NA	NA	NA	NA
				Pac 80 mg/m^2^ d1,8,15 q4w	276	NA	NA	NA	NA	0	0	NA	NA	NA	NA	NA	NA
[23]	II	Melanoma	PFS	Pem 2 mg/kg q3w	178	NA	NA	NA	NA	2	1	NA	NA	NA	NA	NA	NA
				Pem 10 mg/kg q3w	179	NA	NA	NA	NA	2	2	NA	NA	NA	NA	NA	NA
				ICC q3w	171	NA	NA	NA	NA	1	0	NA	NA	NA	NA	NA	NA
[24]	II/III	NSCLC	OS,PFS	Pem 2 mg/kg q3w	339	16	2	10	2	1	1	NA	NA	3	0	4	0
				Pem 10 mg/kg q3w	343	8	1	7	0	2	0	NA	NA	8	2	1	1
				Doc 75 mg/m^2^ q3w	309	4	0	3	0	0	0	NA	NA	2	0	1	1
[25]	III	UC	OS,PFS	Pem 200 mg q3w	266	14	3	14	6	NA	NA	NA	NA	NA	NA	NA	NA
				ICC q3w	255	4	0	3	0	NA	NA	NA	NA	NA	NA	NA	NA
[26]	III	NSCLC	OS	Ave 10 mg/kg q2w	393	7	2	2	NA	4	NA	NA	NA	NA	NA	5	3
				Doc 75 mg/m^2^ q3w	365	3	0	NA	NA	NA	NA	NA	NA	NA	NA	5	2

*Pts*, patients; *ALT*, alanine aminotransferase; *AST*, aspartate aminotransferase; *BIL*, bilirubin; *ALP*, alkaline phosphatase; *GGT*, gamma-glutamyltransferase; *OS*, overall survival; *ORR*, objective response rate; *Niv*, nivolumab; *ICC*, investigator's choice of chemotherapy; *NA*, not available; *NSCLC*, non-small cell lung cancer; *Doc*, docetaxel; *Dac*, dacarbazine; *PFS*, progression-free survival; *Pem*, pembrolizumab; *GC/GEJC*, gastric or gastro-oesophageal junction cancer; *Pac*, Paclitaxel; *UC*, urothelial cancer; *Ave*, avelumab

**Table 2:** Characteristics of the 7 randomized controlled trials that compared PD-1/PD-L1 inhibitors plus chemotherapy vs*.* chemotherapy

Reference	Trial Phase	Tumor type	Primary Endpoint	Treatment arms	Pts	Increased ALT, n		Increased AST, n		Hepatitis, n	
						G1-5	G 3-5	G1-5	G3-5	G1-5	G3-5
[27]	III	Non-squamous NSCLC	OS,PFS	Pem 200 mg + Platinum-based ICC q3w	405	49	2	NA	NA	5	4
				Placebo+ Platinum-based ICC q3w	202	18	3	NA	NA	0	0
[28]	II	Non-squamous NSCLC	ORR	Pem 200 mg + Chemo (Car+pemetrexed) q3w	59	10	1	11	1	NA	NA
				Chemo (Car+pemetrexed) q3w	62	7	1	7	1	NA	NA
[29]	III	Squamous-cell NSCLC	OS,PFS	Pem 200 mg + Chemo (Car+[nab-]pac) q3w	278	NA	NA	NA	NA	5	5
				Placebo + Chemo (Car+[nab-]pac) q3w	280	NA	NA	NA	NA	0	0
[30]	III	ES-SCLC	OS,PFS	Ate 1200 mg + Chemo (Car+Eto) q3w	198	NA	NA	NA	NA	14	3
				Placebo + Chemo (Car+Eto) q3w	196	NA	NA	NA	NA	9	0
[31]	III	Triple-negative BC	OS,PFS	Ate 840 mg q3w + nab-pac 100 mg/m2 d1,8,15 q4w	452	47	8	NA	NA	10	6
				Placebo+ nab-pac 100 mg/m2 d1,8,15 q4w	438	40	5	NA	NA	7	1
[32]	III	First-line NSCLC	PFS	Ate 1200 mg + Bev + Car+Pac q3w	393	21	4	20	4	8	4
				Bev 15 mg/kg + Car AUC 6+ Pac 175-200 mg/m^2^ q3w	394	13	1	9	1	0	0
[33]	III	Non-squamous NSCLC	OS,PFS	Ate 1200 mg + CarAUC6 q3w + nab-pac100mg/m^2^ qw	473	25	6	17	6	8	2
				Car AUC 6 q3w + nab-pac 100 mg/m^2^ qw	232	14	4	9	3	3	1

*Chemo*, chemotherapy; *Car*, carboplatin; *ES-SCLC*, extensive-stage small-cell lung cancer; *Ate*, Atezolizumab; *Eto*, etoposide; *BC*, breast cancer; *Bev*, bevacizumab; *AUC*, area under the curve

### Hepatotoxicities: PD-1/PD-L1 inhibitor vs. chemotherapy

#### All- and high-grade elevated hepatic enzymes (ALT, AST, BIL, ALP, and GGT)

**Fig. 1:**
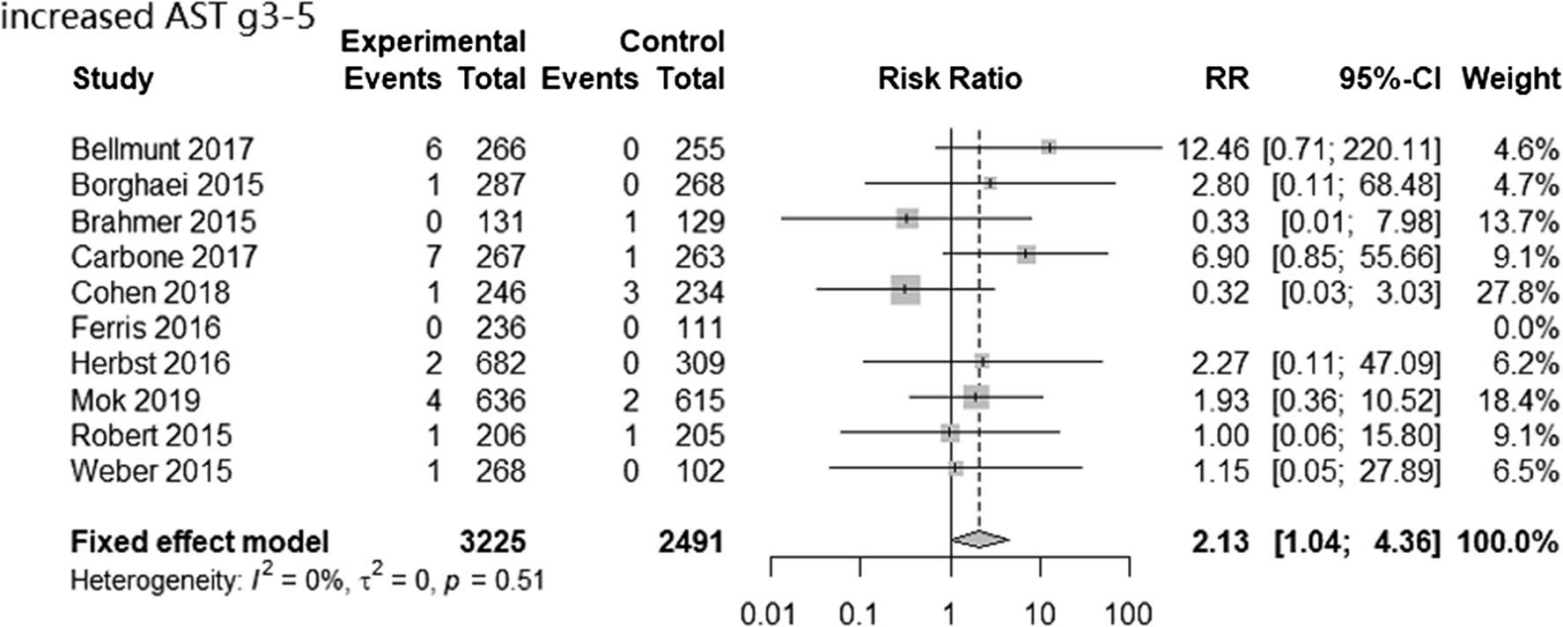
Forest plot for high-grade AST elevation in studies that compared PD-1/PD-L1 inhibitors *vs.* chemotherapy

The PD-1/PD-L1 inhibitor and chemotherapy groups had no significant differences in RR for all-grade elevated ALT, AST, BIL, ALP, and GGT levels and no significant differences for high-grade elevated ALT, BIL, ALP, and GGT levels (Table 3 and Supplementary Figure S3), but the PD-1/PD-L1 inhibitor group had a greater RR for high-grade elevated AST (RR = 2.13, 95% CI = 1.04 to 4.36, *p* = 0.04; Fig. 1). However, the RR of high-grade elevated AST was not significantly different when comparing the pembrolizumab and nivolumab subgroups *vs.* chemotherapy (pembrolizumab: RR = 2.04, 95%CI = 0.78 to 5.32, P = 0.15; nivolumab: RR = 2.25, 95%CI = 0.76 to 6.63, P = 0.14; Supplementary Figure S4).

**Table 3:** The risk estimates that compared PD-1/PD-L1 inhibitors *vs.* chemotherapy

AE type	RR (95%CL), *P* value	
	Grade 1–5	Grade 3–5
Increased ALT	1.19 (0.92–1.54), 0.18	1.58 (0.88–2.83), 0.12
Increased AST	1.33 (1.01–1.76), 0.05	2.13 (1.04–4.36), 0.04
Increased BIL	0.88 (0.31–2.44), 0.8	2.85 (0.12–69.71), 0.52
Increased ALP	0.97 (0.42–2.26), 0.95	0.91 (0.13–6.46), 0.93
Increased GGT	1.04 (0.46–2.36), 0.92	1.39 (0.52–3.75), 0.51

#### All- and high-grade hepatitis

**Fig. 2:**
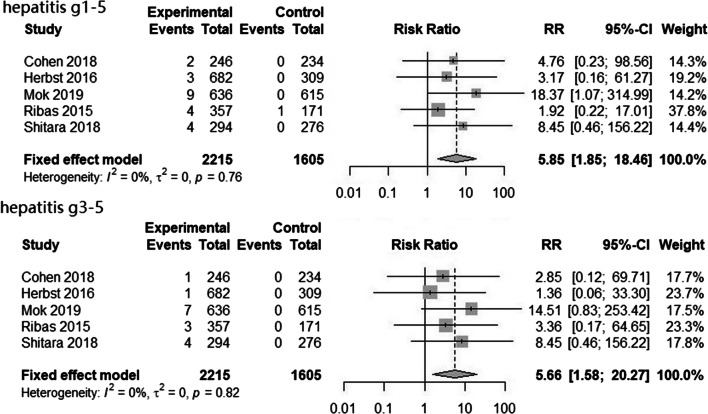
Forest plot for hepatitis in studies that compared PD-1/PD-L1 inhibitors vs. chemotherapy

When comparing pembrolizumab vs. chemotherapy, there was a significant increase in the RR of all-grade hepatitis (RR = 5.85, 95% CI = 1.85 to 18.46, *P* < 0.01) and high-grade hepatitis (RR = 5.66, 95% CI = 1.58 to 20.27, *P* < 0.01; Fig. 2).

### Hepatotoxicities: PD-1/PD-L1 inhibitor plus chemotherapy vs. chemotherapy

#### All- and high-grade elevated hepatic enzymes (ALT, AST)

The two groups had no significant differences in RR for all-grade and high-grade elevated ALT and all-grade and high-grade elevated AST (Table 4 and Supplementary Figure S5).

**Table 4:** The risk estimates that compared PD-1/PD-L1 inhibitors plus chemotherapy vs. chemotherapy

.AE type	RR (95%CL), *P* value	
	Grade 1–5	Grade 3–5
Increased ALT	1.23 (0.96–1.57), 0.11	1.12 (0.58–2.12), 0.176
Increased AST	1.52 (0.96–2.42), 0.07	1.50 (0.54–4.18), 0.44

#### All- and high-grade hepatitis

**Fig. 3:**
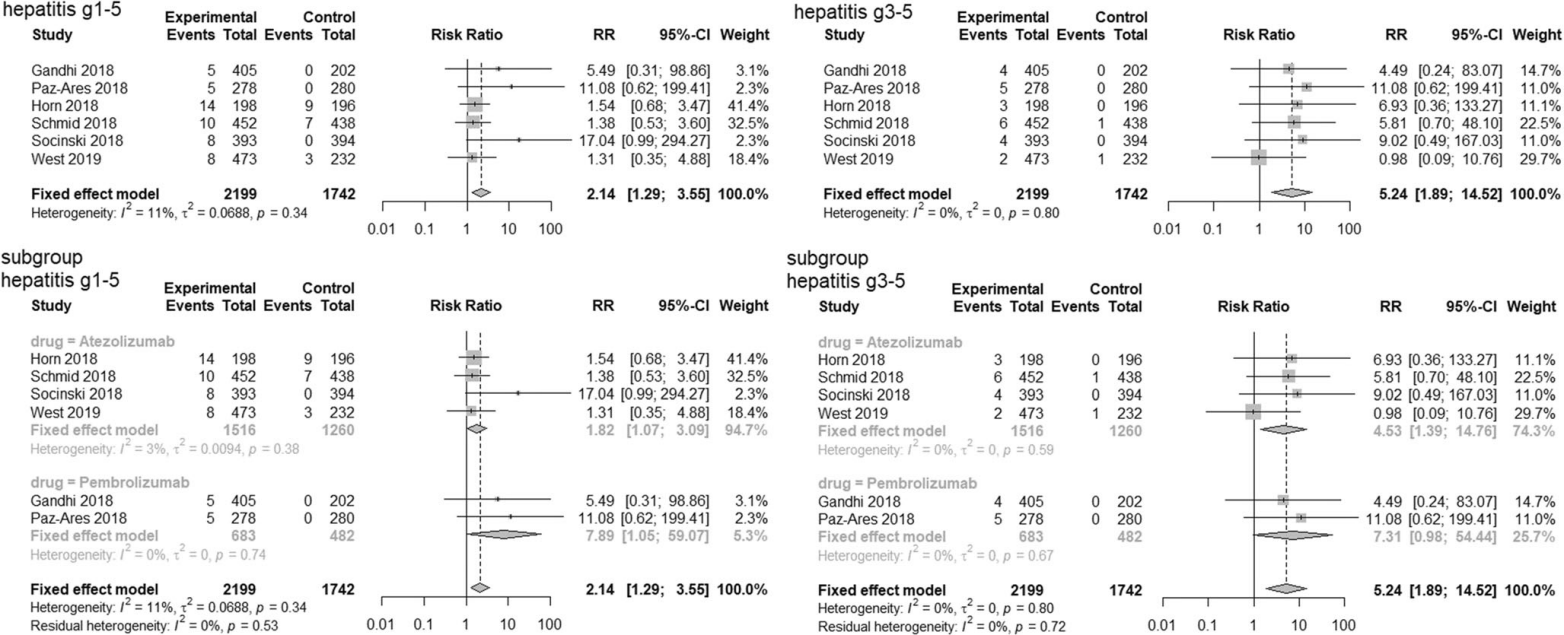
Forest plots for hepatitis in studies and subgroup analysis of different PD-1/PD-L1 inhibitors plus chemotherapy vs. chemotherapy alone

Compared with patients treated with chemotherapy alone, those treated with a PD-1/PD-L1 inhibitor plus chemotherapy were more likely to experience all-grade hepatitis (RR = 2.14, 95% CI = 1.29 to 3.55, *P* < 0.01) and high-grade hepatitis (RR = 5.24, 95% CI = 1.89 to 14.52, *P* < 0.01). However, subgroup analysis showed that pembrolizumab plus chemotherapy only marginally increased the risk of high-grade hepatitis (RR = 7.31, 95% Cl = 0.98 to 54.44, *P* = 0.05) but that atezolizumab plus chemotherapy significantly increased the RR of high-grade hepatitis (RR = 4.53, 95% Cl = 1.39 to 14.76, *P* = 0.01). Comparison of the two combined treatments (atezolizumab plus chemotherapy vs. pembrolizumab plus chemotherapy) indicated no significant difference in the RR for high-grade hepatitis (*P* = 0.69). Analysis of all-grade hepatitis indicated a greater RR in the pembrolizumab subgroup (RR = 7.89, 95% CI = 1.05 to 59.07, *P* = 0.04) and the atezolizumab subgroup (RR = 1.82, 95% CI = 1.07 to 3.09, *P* = 0.03). As above, comparison of the two combined treatments indicated no significant difference in the RR for all-grade hepatitis (*P* = 0.17; Fig. 3).

### Deaths related to hepatotoxicity

Only two studies reported deaths due to treatment-related hepatotoxicity. Schmid et al. [31] reported that one patient treated with atezolizumab plus nab-paclitaxel died from autoimmune hepatitis and another patient treated with placebo plus nab-paclitaxel died from hepatic failure. West et al. [33] reported one death from hepatic cirrhosis following treatment with atezolizumab plus chemotherapy.

### Quality assessment and publication bias

We used a fixed effects model for all comparisons due to the low heterogeneity among the included studies. The results of Begg’s test and Egger’s test indicated no evidence of publication bias (Supplementary Table S3).

## Discussion

Hepatotoxicity following ICI treatment is an uncommon but clinically crucial and potentially life-threatening AE. Although hepatocellular or mixed hepatitis with immune features generally occurs within 2 to 12 weeks following initiation of ICI treatment, it may occur at any time throughout treatment, even 1 year after the first dose. Many cases are asymptomatic or mild and require little to no intervention. However, some cases are anicteric initially, but then worsen and become life-threatening without proper intervention [34, 35]. Therefore, the American Society of Clinical Oncology (ASCO) recommends that when a grade 1 liver function test elevation occurs in a patient receiving an ICI, the patient should be followed up with regular liver examinations (measurements of AST, ALT, and BIL) before each dose and/or weekly [36]. In practice, oncologists should be aware of the severity and changes in the severity of AEs. Upon identification of a patient with advanced liver damage or failure, the National Comprehensive Cancer Network (NCCN) recommends discontinuation of immunotherapy and initiation of systemic corticosteroids [37].

The negative effects of different ICIs on hepatic function remain controversial. A 2015 meta-analysis [5] reported no statistically significant difference between CTLA-4 and PD-1 inhibitors in a subgroup analysis, but a 2017 meta-analysis [6] concluded that CTLA-4 inhibitors were more likely to cause hepatotoxicity than PD-1 inhibitors. Additionally, the 2015 meta-analysis indicated a causal relationship of ICI treatment with high-grade elevated transaminase levels, but the 2017 meta-analysis showed that ICI treatment was associated with a higher risk of all-grade and high-grade hepatotoxicity. There may be several reasons for these different findings. First, these two meta-analyses used different baseline and control groups. Specifically, the 2015 study compared chemotherapy with PD-1 inhibitors, whereas the 2017 study compared CTLA-4 inhibitors with several treatment regimens (placebo, dual use of ICIs, and vaccines). Second, both meta-analyses were limited by the small sample sizes of the targeted subgroups. In particular, for the 2015 study, the subgroup analysis of all-grade elevated ALT following PD-1 inhibitor vs. chemotherapy only examined 3 articles. For the 2017 study, we are unaware of any protocol designed to examine hepatotoxicity following CTLA-4 inhibitor monotherapy vs. chemotherapy.

Our findings bridge a gap in knowledge by comparing cancer patients’ risk of hepatotoxicity following PD-1/PD-L1 inhibitors with or without chemotherapy vs. chemotherapy alone. All of the 20 included studies had at least one control arm that received standard chemotherapy alone. Our results indicated little or no difference in risk of elevated blood indicators of hepatotoxicity for patients who received PD-1/PD-L1 inhibitor monotherapy, a PD-1/PD-L1 inhibitor plus chemotherapy, and chemotherapy alone. Although we found a significant overall increase in the RR of high-grade elevated AST with the use of PD-1/PD-L1 inhibitor rather than standard chemotherapy, the significance was borderline (*P* = 0.04), and our 95% CI was wider than reported by Bellmunt et al. [25]. Furthermore, removal of the Bellmunt et al. study from our meta-analysis led to no significant difference in the risk of high-grade elevated AST. Thus, the finding of statistical significance was not stable.

There are reports of higher incidences of discontinuing ICI treatment because of significant hepatotoxicity in patients receiving sunitinib or pazopanib with nivolumab for treatment of renal cell carcinoma (RCC) [38] and using ipilimumab with BRAF inhibitors for treatment of melanoma [39]. These findings may be explained by the toxic effects of the antiangiogenic drugs rather than ICIs. Hence, clinical oncologists should carefully consider use of antiangiogenic drugs and their doses when combining them with an ICI to improve hepatic safety.

Our meta-analysis showed that, compared with standard chemotherapy, PD-1/PD-L1 inhibitor monotherapy and PD-1/PD-L1 inhibitor plus chemotherapy significantly increased the risk of all-grade and high-grade hepatitis. We found no descriptions of hepatitis in any of the trials that examined nivolumab, but some case reports indicated immune-related hepatitis after nivolumab treatment [35, 40].

We noticed that the elevated blood markers of hepatotoxicity in these RCTs could be recorded for any reason, such as immune-related liver injury or drug toxicity. Although the reasons are different, PD-1/PD-L1 inhibitors and chemotherapy have similar probabilities of causing elevated hepatic enzymes. However, investigators of these RCTs focused on immune-mediated AEs, so all cases of hepatitis are immune-related, not on toxic drug effects. Thus, it is more precise to say PD-1/PD-L1 inhibitors increased the risk of immune-related hepatitis. Unfortunately, there is no universally accepted definition of immune-related hepatitis in the clinical literature. As a result, it is difficult to distinguish immune-related hepatitis from other etiologies of liver damage. All types of liver damage are generally characterized by fatigue, loss of appetite, and asymptomatic increases of ALT, AST, and sometimes total BIL. However, based on histopathological findings, it is very rare for patients using PD-1/PD-L1 inhibitors to exhibit signs of liver damage. Moreover, patients with immune-mediated hepatitis generally do not have symptoms of autoimmune hepatitis, namely, plasma cell infiltration, severe interface hepatitis, piecemeal necrosis, and rosette formation. Therefore, by first excluding these causes of induced and autoimmune hepatitis, oncologists may be better able to diagnose immune-related hepatitis by consideration of the therapeutic regimen, laboratory tests, and a liver biopsy.

Hoofnagle et al. [4] classified drug-induced liver injury as direct, indirect, or idiosyncratic hepatotoxicity. Specifically, indirect hepatotoxicity is caused by a drug’s effects on the liver, rather than by its inherent toxicity or idiosyncratic properties (immunogenicity). This is exactly the case for ICIs, because these drugs activate T cells against tumor cells, and the activated T cells then attack normal tissues, resulting in immune-related toxicity in liver [3]. This is similar to the effect of various immunomodulatory agents, such as antineoplastic checkpoint inhibitors, which can also lead to immune-mediated liver injury. These agents are also responsible for indirect drug-induced liver injury, because they are monoclonal antibodies and are thus unlikely to cause direct or idiosyncratic liver injury [4].

Although immune-induced liver injury and autoimmune liver disorders can have a similar pathogeneses, they are actually different disease entities. In particular, Zen et al. [41] compared the clinicopathologic symptoms of ICI-induced liver injury and acutely presenting autoimmune hepatitis or idiosyncratic drug-induced liver injury. They found that patients with ICI-induced liver injury had no antinuclear antibodies or IgG elevations and that hyper-bilirubinemia was less common than elevated liver enzymes. Although the extent of portal inflammation and lobular injury did not significantly differ among ICI-induced, autoimmune, and idiosyncratic drug-induced hepatitis, there were some notable differences. In particular, centrilobular confluent necrosis and plasmacytosis in ICI-induced liver injury were markedly less common and milder than in autoimmune hepatitis; liver injury caused by immunotherapy was associated with markedly fewer CD20+ and CD4+ lymphocytes than autoimmune hepatitis; and eosinophilic infiltration was less common and there were fewer CD20+ and CD4+ lymphocytes in immunotherapy-triggered hepatitis than in idiosyncratic drug-induced liver injury. The differences in lymphocyte subsets may be because PD-1 and CTLA4 are mostly expressed by CD8+ cytotoxic T lymphocytes, and interactions between CD4+ helper T cells and B cells may be less activated in immunotherapy-associated liver injury than in the other types of liver injury.

Anti-PD-1/PD-L1 and anti-CTLA-4 monoclonal antibodies (mAbs) can lead to different pathological features of immunotherapy-associated liver damage. In fact, a histological study by De Martin et al. [35] reported a relationship of anti-CTLA-4 mAbs with granulomatous hepatitis, including fibrin ring granulomas and central vein endotheliitis. However, to the best of our knowledge, the histological pattern in anti-PD-1/PD-L1 hepatitis is characterized by lobular and non-granulomatous hepatitis. Although the histological responses to PD-1/PD-L1 mAbs are diverse, there is a lower incidence of liver irAEs to anti-PD-1/PD-L1 agents than anti-CTLA-4 mAbs, and few records of PD-1/PD-L1 inhibitor-induced hepatitis are available. Corticosteroid therapy is the mainstay of irAE management, although the response of immune-related liver injury to corticosteroid therapy can be variable. Kopecky et al. [40] demonstrated a poor response to corticosteroid therapy when cholestatic hepatitis occurred in immune-mediated liver injury, and suggested use of a more potent immunosuppressive treatment.

The results indicated that PD-1/PD-L1 with or without chemotherapy did not increase the risk of elevated blood markers of hepatotoxicity. On the whole, we think this regimen is safe in clinical practice, although there is little known about immune-related hepatitis. Furthermore, atezolizumab and nab-paclitaxel could increase the risk of several critical clinical endpoints, so we believe that there is need for more research on iAEs to more thoroughly assess the safety of these drugs.

Administration of chemotherapy or immunosuppressive/biologic therapy to a subject with an inactive chronic or resolved hepatitis B virus (HBV) infection (anti-HBc+) can trigger HBV reactivation (defined as the abrupt reappearance or increase of HBV DNA in the serum of a patient with previously inactive or resolved HBV infection) [42]. As far as we know, ICI-induced HBV reactivation is unlikely, and there are no specific indications for the management of these subjects receiving ICIs in the guidelines of the European Association for the Study of the Liver (EASL) and the American Association for the Study of Liver Diseases (AASLD) [42, 43]. Similarly, it should be safe to administer ICIs to treat cancer patients with concurrent chronic hepatitis C. Actually, there are no reported cases of HCV-related flares recorded in any of the ICI trials. Moreover, several studies [44, 45] reported that by increasing the activities of intrahepatic CD4+ and CD8+ T cells, anti-PD-1 agents decreased HCV viremia. In contrast, nivolumab-related hepatotoxicity is similar in patients with and without hepatocellular carcinoma (HCC) [46].

There were several limitations in this meta-analysis. First, due to the lack of hepatic AEs reported in many studies, there were only a limited number of eligible publications. Moreover, because of the heterogeneity of the selected RCTs, namely different diagnostic criteria, researchers, and organizations, the identification of immune-related hepatotoxicity may not be completely consistent. Second, we did not have access to individual patient information or long-term follow-up data of patients, so we did not consider the details of immune-related hepatotoxicity. Similarly, although patients with advanced tumors are generally prone to hepatic metastases, the lack of patient-level information limited our ability to identify the specific abnormalities among patients with liver irAEs. Lastly, all included studies excluded patients with known autoimmune diseases, so the safety of PD-1/PD-L1 inhibitors for patients with pre-existing autoimmune diseases remains unknown.

## Conclusion

Our results indicated that ICIs have favorable hepatic safety profiles and thus support efforts by clinicians to identify patients who are most likely to benefit from PD-1/PD-L1 inhibitor monotherapy and the ongoing development of PD-1/PD-L1 inhibitors in combination therapy regimens. However, the infrequent irAEs in patients using ICIs should not be ignored. Clinicians must remain aware of abnormal liver function, especially immune-related hepatitis, when using PD-1/PD-L1 inhibitors to treat patients with solid tumors so that these patients can be managed appropriately. Importantly, we found no significant differences in the risk of elevated blood indicators of hepatotoxicity for patients receiving PD-1/PD-L1 inhibitor monotherapy, PD-1/PD-L1 inhibitor plus chemotherapy, or chemotherapy alone.

## Electronic supplementary material

ESM 1 (DOCX 2467 kb)

ESM 2 (DOC 62 kb)

## References

[1] Bray F, Ferlay J, Soerjomataram I, Siegel RL, Torre LA, Jemal A (2018). Global cancer statistics 2018: GLOBOCAN estimates of incidence and mortality worldwide for 36 cancers in 185 countries. CA Cancer J Clin.

[2] Hoos A (2016). Development of immuno-oncology drugs - from CTLA4 to PD1 to the next generations. Nat Rev Drug Discov.

[3] Postow MA, Sidlow R, Hellmann MD (2018). Immune-related adverse events associated with immune checkpoint blockade. N Engl J Med.

[4] Hoofnagle JH, Bjornsson ES (2019). Drug-induced liver injury - types and phenotypes. N Engl J Med.

[5] Abdel-Rahman O, ElHalawani H, Fouad M (2015). Risk of elevated transaminases in cancer patients treated with immune checkpoint inhibitors: a meta-analysis. Expert Opin Drug Saf.

[6] Wang W, Lie P, Guo M, He J (2017). Risk of hepatotoxicity in cancer patients treated with immune checkpoint inhibitors: a systematic review and meta-analysis of published data. Int J Cancer.

[7] Green JPHS (2011) Cochrane Handbook for Systematic Reviews of Interventions Version 5.1.0 The Cochrane Collaboration. http://handbook-5-1.cochrane.org/

[8] Moher D, Liberati A, Tetzlaff J, Altman DG, Group P (2009). Preferred reporting items for systematic reviews and meta-analyses: the PRISMA statement. BMJ.

[9] Huang X, Lin J, Demner-Fushman D (2006) Evaluation of PICO as a knowledge representation for clinical questions. AMIA Annu Symp Proc:359–363 PMC1839740 17238363

[10] Institute NC Common Terminology Criteria for Adverse Events (CTCAE) Version4.0.2010. https://evs.nci.nih.gov/ftp1/CTCAE/CTCAE_4.03/CTCAE_4.03_2010-06-14_QuickReference_5x7.pdf

[11] Higgins JP, Altman DG, Gøtzsche PC (2011). The Cochrane Collaboration's tool for assessing risk of bias in randomised trials. Bmj.

[12] Higgins JP, Thompson SG, Deeks JJ, Altman DG (2003). Measuring inconsistency in meta-analyses. Bmj.

[13] DerSimonian R, Laird N (2015). Meta-analysis in clinical trials revisited. Contemp Clin Trials.

[14] Weber JS, D'Angelo SP, Minor D (2015). Nivolumab versus chemotherapy in patients with advanced melanoma who progressed after anti-CTLA-4 treatment (CheckMate 037): a randomised, controlled, open-label, phase 3 trial. Lancet Oncol.

[15] Borghaei H, Paz-Ares L, Horn L (2015). Nivolumab versus docetaxel in advanced nonsquamous non-small-cell lung cancer. N Engl J Med.

[16] Brahmer J, Reckamp KL, Baas P (2015). Nivolumab versus docetaxel in advanced squamous-cell non-small-cell lung cancer. N Engl J Med.

[17] Carbone DP, Reck M, Paz-Ares L (2017). First-line nivolumab in stage IV or recurrent non-small-cell lung cancer. N Engl J Med.

[18] Ferris RL, Blumenschein G (2016). Nivolumab for recurrent squamous-cell carcinoma of the head and neck. N Engl J Med.

[19] Robert C, Long GV, Brady B (2015). Nivolumab in previously untreated melanoma without BRAF mutation. N Engl J Med.

[20] Cohen EEW, Soulières D, Le Tourneau C (2019). Pembrolizumab versus methotrexate, docetaxel, or cetuximab for recurrent or metastatic head-and-neck squamous cell carcinoma (KEYNOTE-040): a randomised, open-label, phase 3 study. Lancet.

[21] Mok TSK, Wu Y-L, Kudaba I (2019). Pembrolizumab versus chemotherapy for previously untreated, PD-L1-expressing, locally advanced or metastatic non-small-cell lung cancer (KEYNOTE-042): a randomised, open-label, controlled, phase 3 trial. Lancet.

[22] Shitara K, Özgüroğlu M, Bang Y-J (2018). Pembrolizumab versus paclitaxel for previously treated, advanced gastric or gastro-oesophageal junction cancer (KEYNOTE-061): a randomised, open-label, controlled, phase 3 trial. Lancet.

[23] Ribas A, Puzanov I, Dummer R (2015). Pembrolizumab versus investigator-choice chemotherapy for ipilimumab-refractory melanoma (KEYNOTE-002): a randomised, controlled, phase 2 trial. Lancet Oncol.

[24] Herbst RS, Baas P, Kim D-W (2016). Pembrolizumab versus docetaxel for previously treated, PD-L1-positive, advanced non-small-cell lung cancer (KEYNOTE-010): a randomised controlled trial. Lancet.

[25] Bellmunt J, de Wit R, Vaughn DJ (2017). Pembrolizumab as second-line therapy for advanced urothelial carcinoma. N Engl J Med.

[26] Barlesi F, Vansteenkiste J, Spigel D (2018). Avelumab versus docetaxel in patients with platinum-treated advanced non-small-cell lung cancer (JAVELIN Lung 200): an open-label, randomised, phase 3 study. Lancet Oncol.

[27] Gandhi L, Rodriguez-Abreu D, Gadgeel S (2018). Pembrolizumab plus chemotherapy in metastatic non-small-cell lung cancer. N Engl J Med.

[28] Langer CJ, Gadgeel SM, Borghaei H (2016). Carboplatin and pemetrexed with or without pembrolizumab for advanced, non-squamous non-small-cell lung cancer: a randomised, phase 2 cohort of the open-label KEYNOTE-021 study. Lancet Oncol.

[29] Paz-Ares L, Luft A, Vicente D (2018). Pembrolizumab plus chemotherapy for squamous non–small-cell lung cancer. N Engl J Med.

[30] Horn L, Mansfield AS, Szczesna A (2018). First-line atezolizumab plus chemotherapy in extensive-stage small-cell lung cancer. N Engl J Med.

[31] Schmid P, Adams S, Rugo HS (2018). Atezolizumab and nab-paclitaxel in advanced triple-negative breast cancer. N Engl J Med.

[32] Socinski MA, Jotte RM, Cappuzzo F (2018). Atezolizumab for first-line treatment of metastatic nonsquamous NSCLC. N Engl J Med.

[33] West H, McCleod M, Hussein M (2019). Atezolizumab in combination with carboplatin plus nab-paclitaxel chemotherapy compared with chemotherapy alone as first-line treatment for metastatic non-squamous non-small-cell lung cancer (IMpower130): a multicentre, randomised, open-label, phase 3 trial. Lancet Oncol.

[34] Huffman BM, Kottschade LA, Kamath PS, Markovic SN (2018). Hepatotoxicity after immune checkpoint inhibitor therapy in melanoma: natural progression and management. Am J Clin Oncol.

[35] De Martin E, Michot JM, Papouin B (2018). Characterization of liver injury induced by cancer immunotherapy using immune checkpoint inhibitors. J Hepatol.

[36] Friedman CF, Proverbs-Singh TA, Postow MA (2016). Treatment of the immune-related adverse effects of immune checkpoint inhibitors. JAMA Oncol.

[37] Lleo A, Rimassa L, Colombo M (2019). Hepatotoxicity of immune check point inhibitors: Approach and management. Dig Liver Dis.

[38] Amin A, Plimack ER, Ernstoff MS (2018). Safety and efficacy of nivolumab in combination with sunitinib or pazopanib in advanced or metastatic renal cell carcinoma: the CheckMate 016 study. J Immunother Cancer.

[39] Ribas A, Hodi FS, Callahan M (2013). Hepatotoxicity with combination of vemurafenib and ipilimumab. N Engl J Med.

[40] Kopecky J, Kubecek O, Geryk T (2019). Hepatic injury induced by a single dose of nivolumab - a case report and literature review. Klin Onkol.

[41] Zen Y, Yeh MM (2018). Hepatotoxicity of immune checkpoint inhibitors: a histology study of seven cases in comparison with autoimmune hepatitis and idiosyncratic drug-induced liver injury. Mod Pathol.

[42] Terrault NA, Lok ASF, McMahon BJ (2018). Update on prevention, diagnosis, and treatment of chronic hepatitis B: AASLD 2018 hepatitis B guidance. Hepatology.

[43] EASL (2017). Clinical Practice Guidelines on the management of hepatitis B virus infection (2017). J Hepatol.

[44] Fuller MJ, Callendret B, Zhu B (2013). Immunotherapy of chronic hepatitis C virus infection with antibodies against programmed cell death-1 (PD-1). Proc Natl Acad Sci U S A.

[45] Gardiner D, Lalezari J, Lawitz E (2013). A randomized, double-blind, placebo-controlled assessment of BMS-936558, a fully human monoclonal antibody to programmed death-1 (PD-1), in patients with chronic hepatitis C virus infection. PLoS One.

[46] El-Khoueiry AB, Sangro B, Yau T (2017). Nivolumab in patients with advanced hepatocellular carcinoma (CheckMate 040): an open-label, non-comparative, phase 1/2 dose escalation and expansion trial. Lancet.

