# Supplementary material for: Hepatotoxicity in patients with solid tumors treated with PD-1/PD-L1 inhibitors alone, PD-1/PD-L1 inhibitors plus chemotherapy, or chemotherapy alone: systematic review and meta-analysis
Source: Eur J Clin Pharmacol. 2020 Jun 8;76(10):1345–54. doi: 10.1007/s00228-020-02903-2 (PMC7481165; doi:10.1007/s00228-020-02903-2)

**Table S1: search performed in Pubmed and Embase**

| Recent queries in pubmed | |  |
| --- | --- | --- |
| Search | Query | Items found |
| #7 | Search (avelumab) OR MSB0010718C Filters: Clinical Trial | 18 |
| #6 | Search ((durvalumab) OR MEDI4736) OR Imfinzi Filters: Clinical Trial | 20 |
| #5 | Search ((((atezolizumab) OR anti-PDL1) OR MPDL3280A) OR Tecentriq) OR RG7446 Filters: Clinical Trial | 35 |
| #4 | Search (((pembrolizumab) OR lambrolizumab) OR Keytruda) OR MK-3475 Filters: Clinical Trial | 138 |
| #3 | Search ((((Nivolumab) OR Opdivo) OR ONO4538) OR MDX1106) OR BMS936558 Filters: Clinical Trial | 155 |
| #2 | Search PD-L1 Filters: Clinical Trial | 227 |
| #1 | Search PD-1 Filters: Clinical Trial | 334 |

| Recent queries in EMBASE | | |
| --- | --- | --- |
| No. | Query | Results |
| #7 | 'pd 1':ti,ab,kw AND [randomized controlled trial]/lim | 547 |
| #6 | 'pd l1':ti,ab,kw AND [randomized controlled trial]/lim | 654 |
| #5 | (nivolumab:ti,ab,kw OR opdivo:ti,ab,kw OR ono4538:ti,ab,kw OR mdx1106:ti,ab,kw OR bms936558:ti,ab,kw) AND [randomized controlled trial]/lim | 432 |
| #4 | (pembrolizumab:ti,ab,kw OR lambrolizumab:ti,ab,kw OR keytruda:ti,ab,kw OR 'mk 3475':ti,ab,kw) AND [randomized controlled trial]/lim | 388 |
| #3 | (atezolizumab:ti,ab,kw OR 'anti pdl1':ti,ab,kw OR mpdl3280a:ti,ab,kw OR tecentriq:ti,ab,kw OR rg7446:ti,ab,kw) AND [randomized controlled trial]/lim | 216 |
| #2 | (durvalumab:ti,ab,kw OR medi4736:ti,ab,kw OR imfinzi:ti,ab,kw) AND [randomized controlled trial]/lim | 138 |
| #1 | (msb0010718c:ti,ab,kw OR avelumab:ti,ab,kw) AND [randomized controlled trial]/lim | 55 |

**Table S2: the grade of hepatic investigations according to Common Terminology Criteria for Adverse Events (CTCAE Version 4.0)**

| **Adverse event** | **Grade 1** | **Grade 2** | **Grade 3** | **Grade 4** | **Grade 5** |
| --- | --- | --- | --- | --- | --- |
| **Alanine aminotrasferase increased** | >ULN-3.0*ULN | >3.0-5.0*ULN | >5.0-20.0*ULN | >20.0*ULN | — |
| Definition: A finding based on laboratory test results that indicate an increase in the level of alanine aminotrasferase(ALT or SGPT) in the blood specimen. | | | | | |
| **Aspartate aminotransferase increased** | >ULN-3.0*ULN | >3.0-5.0*ULN | >5.0-20.0*ULN | >20.0*ULN | — |
| Definition: A finding based on laboratory test results that indicate an increase in the level of aspartate aminotrasferase(AST or SGOT) in the blood specimen. | | | | | |
| **Alkaline phophatase increased** | >ULN-2.5*ULN | >2.5-5.0*ULN | >5.0-20.0*ULN | >20.0*ULN | — |
| Definition: A finding based on laboratory test results that indicate an increase in the level of alkaline phophatase(ALP) in a blood specimen. | | | | | |
| **GGT increased** | >ULN-2.5*ULN | >2.5-5.0*ULN | >5.0-20.0*ULN | >20.0*ULN | — |
| Definition: A finding based on laboratory test results that indicate higher than normal level of the enzyme gamma-glutamylthransferase in the blood specimen. GGT(gamma-glutamylthransferase) catalyzes the transfer of a gamma glutamyl group from a gamma glutamyl peptide to another peptide, amino acids or water. | | | | | |
| **Blood bilirubin increased** | >ULN-1.5*ULN | >1.5-3.0*ULN | >3.0-10.0*ULN | >10.0*ULN | — |
| Definition: A finding based on laboratory test results that indicate an abnormally high level of bilirubin(BIL) in the blood. Excess bilirubin is associated with jaundice. | | | | | |
| **Hepatitis** | asymptomatic; intervention not indicated | moderate symptoms; medical intervention indicated | symptomatic liver dysfunction; fibrosis by biopsy; compensated cirrohosis; | decompensated liver function (e.g. ascite, coagulopathy, encephalopathy, coma) | death |
| Definition: Hepatitis is swelling and inflammation of the liver. . | | | | | |

**Table S3: Evaluation of publication bias in the meta analysis with Begg’s and Egger's tests (number of trails≥10)**

| **Treatment** | **Parameters** | **Trails** | **Heterogeneity** | | **Begg’s test** | | **Egger’s test** | |
| --- | --- | --- | --- | --- | --- | --- | --- | --- |
|  |  |  | **P** | **I2** | **Z** | **P** | **T** | **P** |
| PD-1/PD-L1 VS Chemo | increased ALT g1-5 | 11 | 0.11 | 36.40% | -0.39 | 0.7 | 1.43 | 0.19 |
|  | increased ALT g3-5 | 11 | 0.63 | 0% | -1.17 | 0.24 | -0.98 | 0.35 |
|  | increased AST g1-5 | 10 | 0.03 | 52.00% | -0.45 | 0.65 | 0.52 | 0.62 |

**Figure S1: Flowchart depicting the RCTs selection process.**


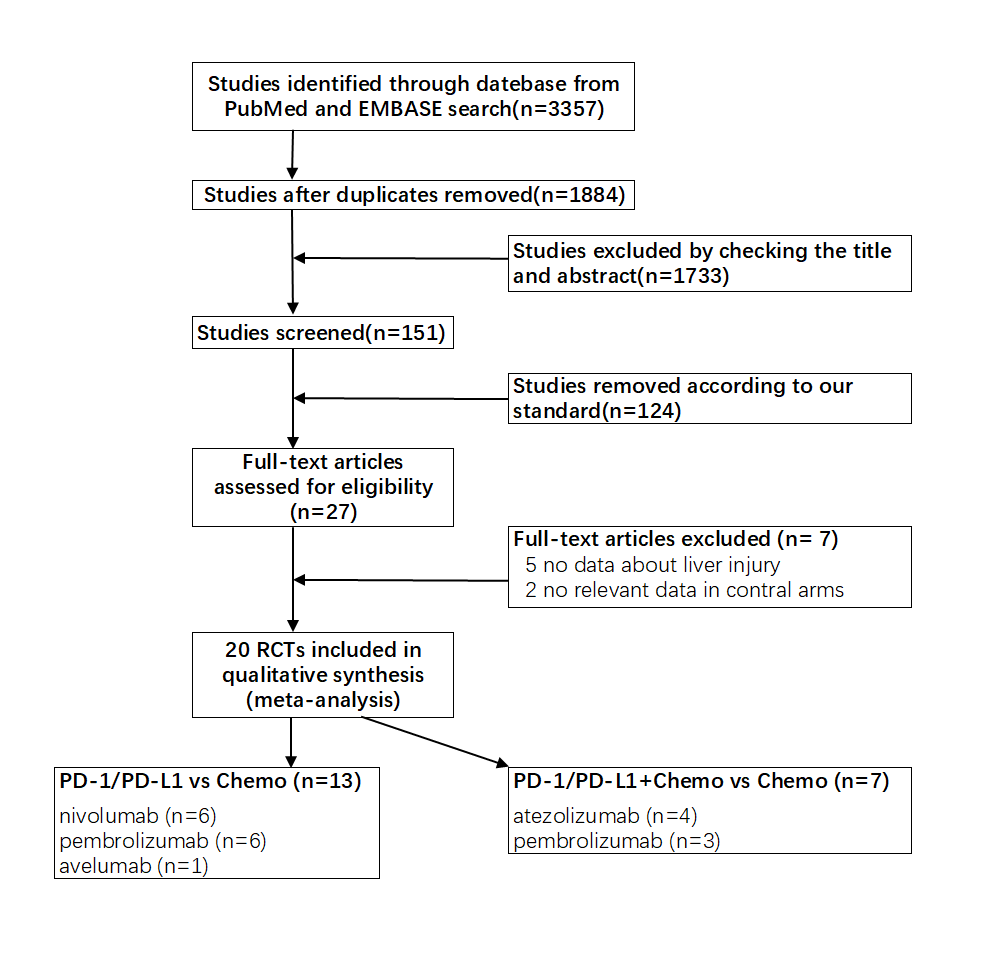


**Figure S2: Risk of bias summary**

**
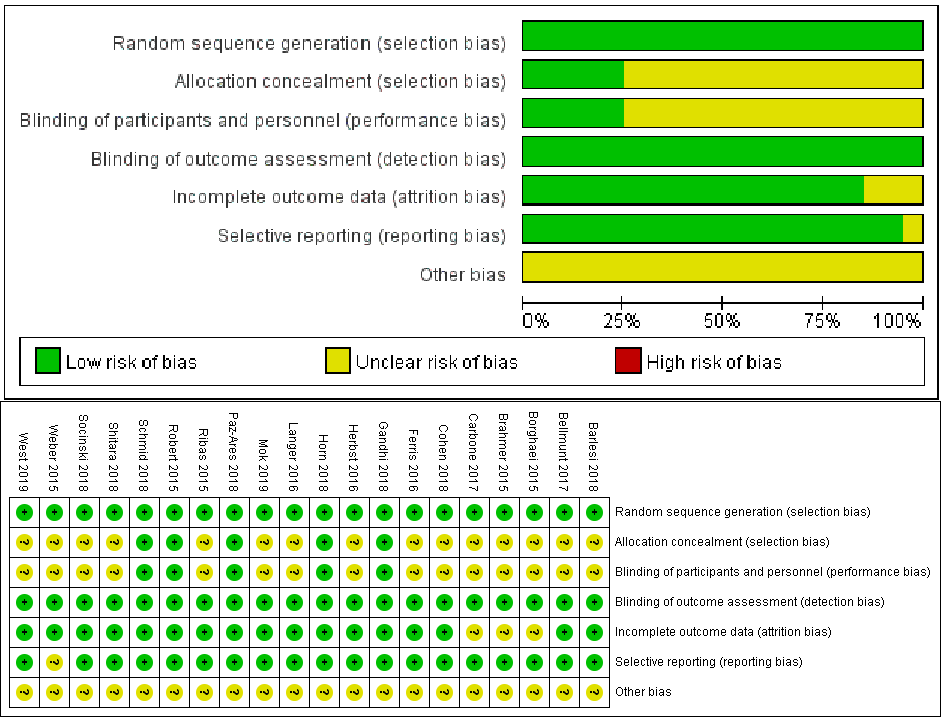
**

(**A**): Bar chart comparing the percentage risk of bias for each included RCT. Low risk of bias (Green), high risk of bias (Red), and unclear risk of bias (Yellow). (**B**): Risk of bias for each included RCT, representing low risk of bias (+), high risk of bias (-), and unclear risk of bias (?).

**Figure S3. Forest plots for hepatotoxicity in studies that compared PD-1/PD-L1 inhibitors *vs.* chemotherapy.**


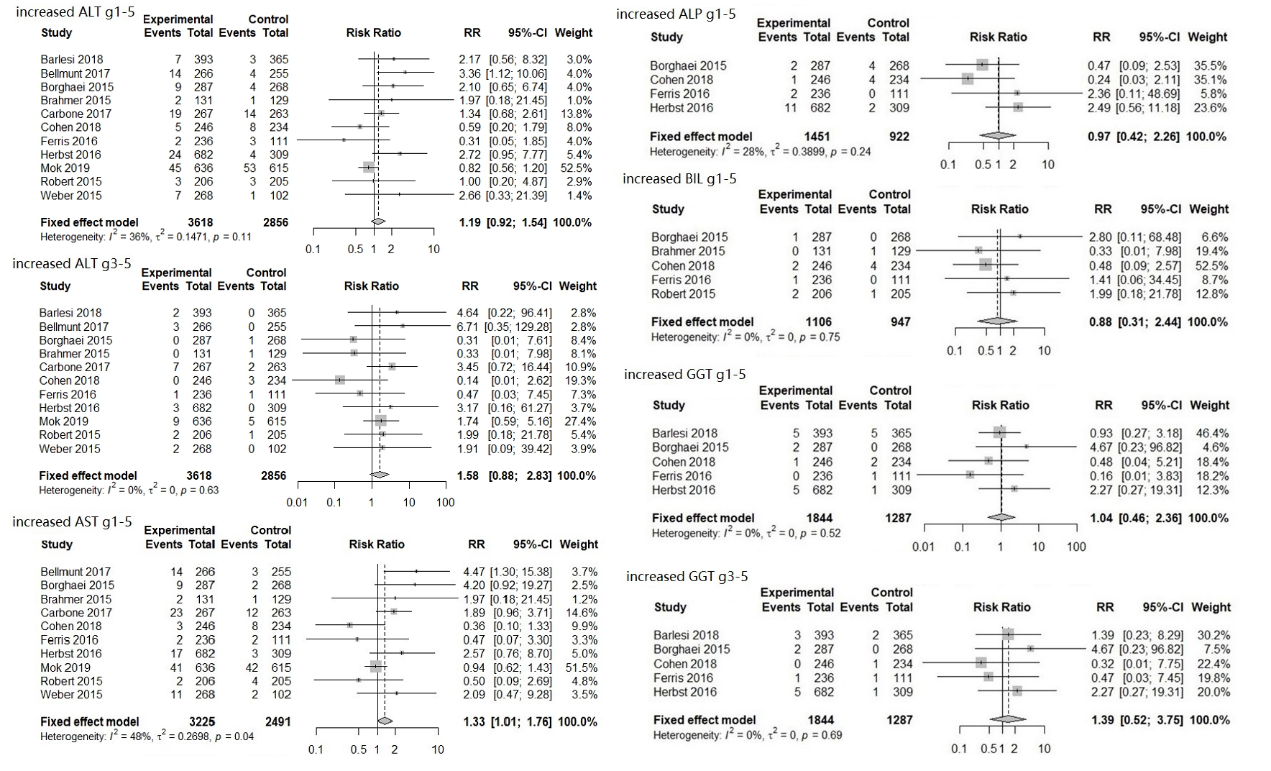


**Figure S4. Forest plots for hepatotoxicity in subgroup analysis of different PD-1/PD-L1 inhibitors *vs.* chemotherapy.**


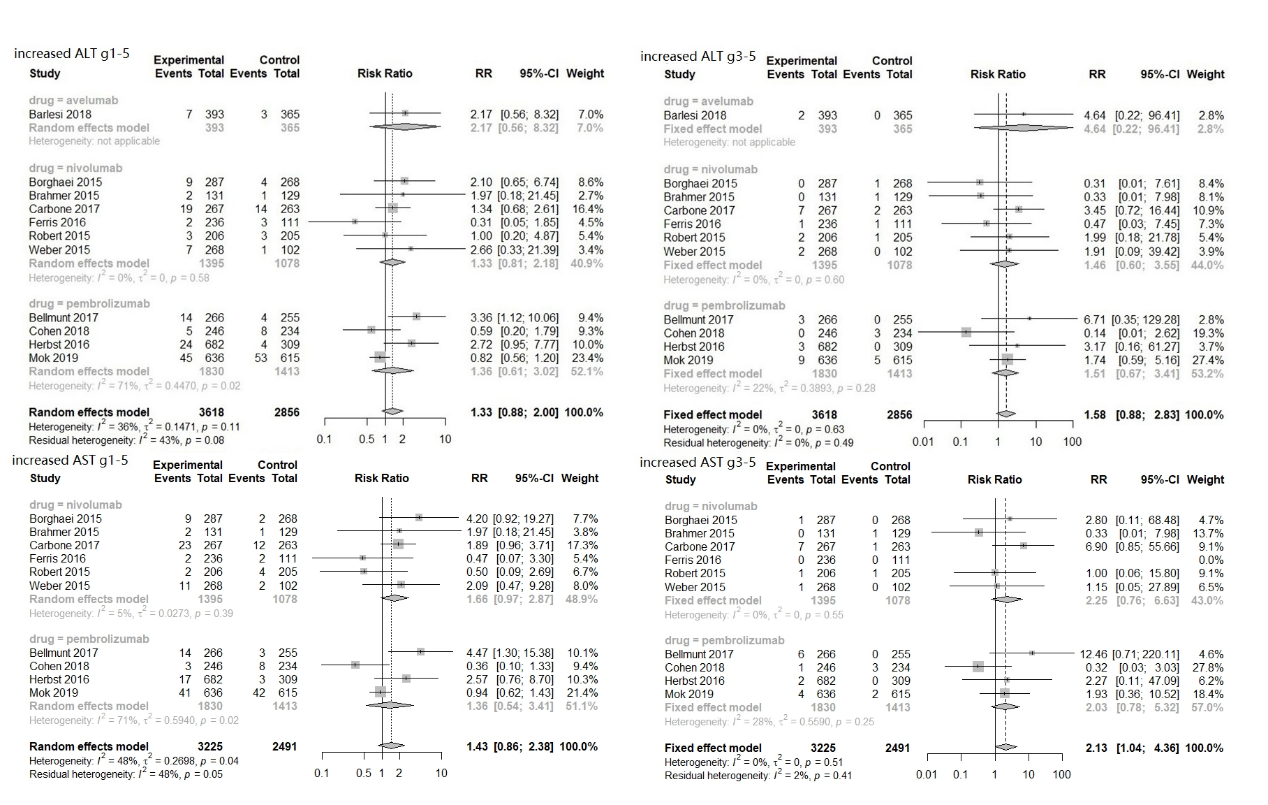


**Figure S5. Forest plots for hepatotoxicity** **in studies that compared PD-1/PD-L1 inhibitors plus chemotherapy *vs.* chemotherapy.**


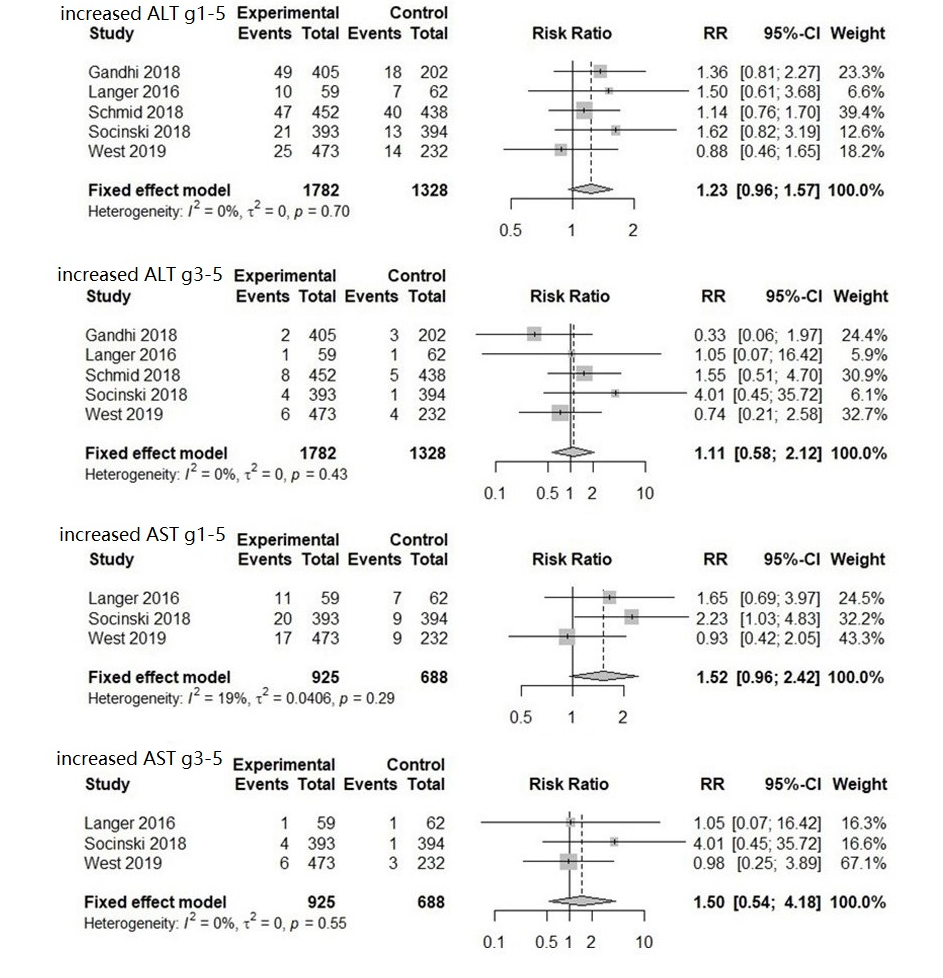

Supplement: Supplementary file 1 — (DOCX 2467 kb) [file 228_2020_2903_MOESM1_ESM.docx]
