# Supplementary material for: Hepatotoxicity in patients with solid tumors treated with PD-1/PD-L1 inhibitors alone, PD-1/PD-L1 inhibitors plus chemotherapy, or chemotherapy alone: systematic review and meta-analysis
Source: Eur J Clin Pharmacol. 2020 Jun 8;76(10):1345–54. doi: 10.1007/s00228-020-02903-2 (PMC7481165; doi:10.1007/s00228-020-02903-2)
Supplement: Supplementary file 2 — (DOC 62 kb) [file 228_2020_2903_MOESM2_ESM.doc]

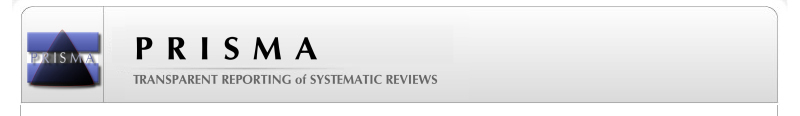
**PRISMA 2009 Flow Diagram**

**Screening**

**Included**

**Eligibility**

**Identification**

3357 potentially relevant articles (PUBMED, n=927; EMBASE, n=2430;)

Additional records identified through other sources
(n = 0 )

Records after duplicates removed
(n =1884)

Records screened
(n =151)

Studies removed according to our standard
(n =124)

Full-text articles assessed for eligibility
(n =27)

7 Full-text articles excluded:
no data about liver injury,n=5

no data in control arm, n=2

Studies included in qualitative synthesis
(n =20)

20 Studies included in quantitative synthesis (meta-analysis)
13 for PD-1/PD-L1 vs Chemo

7 for PD-1/PD-L1+Chemo vs Chemo

Articles excluded after titles/abstracts revision
(n =1733)
